# Efficient Method of Genotyping Ob/Ob Mice Using High Resolution Melting Analysis

**DOI:** 10.1371/journal.pone.0078840

**Published:** 2013-11-13

**Authors:** Alton G. Sutter, Arun P. Palanisamy, Nichole Kurtz, Demetri D. Spyropoulos, Kenneth D. Chavin

**Affiliations:** 1 Division of Transplant Surgery/Department of Surgery, Medical University of South Carolina, Charleston, South Carolina, United States of America; 2 Department of Pathology and Laboratory Medicine, Medical University of South Carolina, Charleston, South Carolina, United States of America; University of Alabama, Birmingham, United States of America

## Abstract

**Objective:**

Direct health care costs of obesity continue to grow throughout the world and research on obesity disease models are on the rise. The ob/ob mouse is a well-characterized model of obesity and associated risk factors. Successful breeding and backcrossing onto different backgrounds are essential to create knockout models. Ob/ob mice are sterile and heterozygotes must be identified by genotyping to maintain breeding colonies. Several methods are employed to detect the ob mutant allele, a single nucleotide polymorphism (SNP). Gel based methods are time consuming and inconsistent, and non-gel based assays rely upon expensive and complex reagents or instruments. A fast, high-throughput, cost effective, and consistent method to identify Lep^ob^ mutation is much needed.

**Design and Methods:**

Primers to produce an amplicon for High Resolution Melting Analysis (HRM) of the Lep^ob^ SNP were designed and validated.

**Results:**

Fluorescence normalized high resolution melting curve plots delineated ob/+, ob/ob, and WT genotypes. Genotypes were also confirmed phenotypically.

**Conclusions:**

HRM of the Lep^ob^ SNP allows closed-tube identification of the Lep^ob^ mutation using a real-time PCR machine now common to most labs/departments. Advantages of this method include assay sensitivity/accuracy, low cost dyes, less optimization, and cost effectiveness as compared to other genotyping techniques.

## Introduction

As the global burden of obesity doubled between 1980 and 2008, the importance of basic science research on the disease grows [1]. Multiple animal models exist for the investigation of obesity. These include diet induced obesity and multi- and poly-genic rodent models. One of the best-characterized and widely used models is the ob/ob mouse, which does not produce functional leptin, yielding hyperphagy, lethargy, and morbid obesity. Ob/ob mice are widely used to study diabetes, fatty liver disease, and leptin signaling. Multiple animal models utilizing the allele have been generated through backcrossing onto different genetic backgrounds (4), and crossing with other mutant strains to create multiple knockout models [2], [3].

Unfortunately, ob/ob mice are sterile and breeding of complete knockouts is impractical as male ob/ob mice must be maintained on a calorie restricted diet, and females supplemented with leptin from an early postnatal age through gestation and nursing [4]. Thus, fertile heterozygotes are identified by genotyping methods to maintain efficient breeding colonies for the production of wild type, heterozygous, and homozygous test animals. Furthermore, heterozygous animals are grossly indistinguishable from wild type, yet deviate in other important phenotypic characteristics (e.g., adiposity, fasting blood glucose, and metabolic efficiency) from homozygous wild type animals. Thus their identification is not only important for breeding purposes, but also to avoid their use in lean control groups [5], [6].

The Lep^ob^ mutation is a single nucleotide polymorphism (SNP) and its detection relies on either gel-based methods that are time consuming and inconsistent or non-gel based assays that rely upon expensive and complex reagents or instrumentation. Restriction fragment length polymorphism (RFLP) analysis methods for the identification of ob/+ heterozygotes were designed by Chung et al, Hirasawa et al, and Namae et al [7], [8], [9]. RFLP analysis is a gel-based method requiring extensive post-PCR processing and the use of restriction enzymes. Other methods such as “pyrosequencing” or end-point analysis require the use of costly instrumentation or fluorescently labeled probes [10], [11]. Further methods using nucleotide invasion assays have been developed which are less labor intensive but require the use of costly probes [10], [12]. Our group previously reported a PCR- based method for ob genotyping [13]. However, this method requires two sets of PCR reactions and gels for each analysis, which is cumbersome and time consuming for the management of large colonies.

High Resolution Melting Analysis (HRM) of the ob SNP, described here, aids in fast, high-throughput, and closed-tube identification of the Lep^ob^ mutation without sacrificing assay sensitivity or accuracy. HRM uses low cost dyes, requires less optimization, is more cost effective than other genotyping techniques due to decreased reagent and personnel costs, and utilizes common RT-PCR instrumentation. HRM analysis has been used to rapidly identify disease causing point mutations and antibiotic resistant bacteria [12], [14], [15], [16].

The first step of the HRM analysis is amplification of the region of interest using standard PCR techniques. The PCR product is then ‘melted’ in the presence of a specialized double-stranded DNA intercalating dye with fluorescence intensity thereby being acquired throughout. The dye is highly fluorescent when bound to dsDNA and poorly fluorescent in the unbound state after dissociation, or ‘melting’, of the DNA duplex. The change in fluorescence, as the DNA is denatured by increasing temperature, is monitored to produce a characteristic melting profile that is sensitive enough to allow the detection of a single base change between otherwise identical nucleotide sequences. HRM analysis is able to discriminate the melting transitions of heteroduplexes by utilizing dyes which are not released at lower temperatures to redistribute and bind to higher melting temperature homoduplexes [13]. It is this discrimination of heteroduplex melting curves that allows the identification of heterozygous animals.

## Methods

### Ethics Statement

Tail clippings from WT, ob/+, and ob/ob mutant mice were collected and utilized for this study because of the nature of information sought. All rodents used for tail clipping were anesthetized using isoflurane. Tails were cauterized after clipping. Animals were observed post-clipping for signs of distress such as discernable pain. Buprenorphine was given as an analgesic drug to reduce pain and discomfort. Animals are removed from the study and euthanized by exsanguination (under anesthesia) or CO2 when suffering negates the need to continue humanely in accordance with the Medical University of South Carolina's Institutional Animal Care and Use Committee (IACUC) policy. This study was reviewed and approved by the Medical University of South Carolina's IACUC (AR# 3003: The Effects of Steatosis on Ischemia/Reperfusion and Liver Regeneration in Mice).

### Animals and Reagents

ob/+ and ob/ob (B6.V-Lepob/J) animals were purchased from Jackson Laboratories (Bar Harbor, ME). All animal practices comply with protocols approved by MUSC IACUC. Primers and ReadyMix Taq Reaction Mix were purchased from Sigma (St Louis, MO) and Direct PCR Lysis Reagent from Viagen (Los Angeles, CA).

### Sample Preparation

Samples were prepared from tails of 8-day-old pups using direct PCR lysis reagent, according to the manufacturer’s protocol. Briefly, 0.5 cm tail snips were collected and digested overnight at 55°C in 0.2 ml reagent containing 50 µg/ml proteinase K. Samples were then purified on DNEasy microcentrifuge slica membranes according to manufacturers protocol and eluted in 0.2 ml buffer (Qiagen, Valencia, CA). Alternately, samples may be heated 45 minutes at 85°C to inactivate proteinase K and PCR performed directly on lysis reaction.

### Primer Design

**Figure 1:**
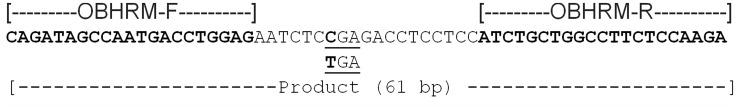
Primers and product around Lep^ob^ SNP that yields premature stop codon.

Forward and reverse primers were chosen to have complimentary annealing temperature and a product length of 61 base pairs centered upon the Lep^ob^ mutation (**Figure 1**), a premature stop codon, at codon 105 of the leptin precursor gene (GenBank # U18812). The relatively short PCR product allows resolution of homozygous melt curves. Predicted PCR product was confirmed by size, via gel electrophoresis, and melting temperature (Tm), using melting temperatures predicted by software (Oligo Calculator version 3.26).

### PCR

PCR was performed using LightCycler 480 High Resolution Melting Master (Roche Diagnostics, Indianapolis, IN) after addition of MgCl_2_ and primers. Final primer concentration of both OBHRM-F and OBHRM-R (**Table 1**) was 0.2 µM. Optimal MgCl_2_ concentration was found to be 2 µM. Sample DNA (0.5 µL elutriate, or approximately 30 ng) was added to 19.5 µL master mix in PCR microplate (USA Scientific, Ocala, FL) wells, bringing the total reaction volume to 20 µL. Amplification and melt curve analysis was then performed on a Roche LightCycler 480 instrument under the following parameters: Detection Format – SYBR Green I, Pre-Incubation –95°C 3 min, Amplification/Quantification – (95°C 30 s, 58°C 30 s, 72°C 1 min w/single acquisition; 38 cycles), High Resolution Melting – (95°C 1 min, 40°C 1 min, 65°C 1 s, 90°C w/25 acquisitions per °C).

**Table 1:** Ob forward and reverse primer sequences for HRM.

Name	Sequence
OBHRM-F	CAGATAGCCAATGACCTGGAG
OBHRM-R	TCTTGGAGAAGGCCAGCAGAT

### Data Interpretation

Fluorescence intensities (Y-axis) of melting curves are normalized within the software. If controls (Ob, Ht, and Wt) are included on the assay plate they can be identified within the software as melting standards, allowing automated calling of genotypes using the “Light Cycler 480 Gene Scanning Software” as described in manufacturer’s protocol. Otherwise, genotypes can be interpreted visually from inspection of melt curves as described in results.

## Results

### Identification of Mutants by High Resolution Melt Curve Analysis

**Figure 2:**
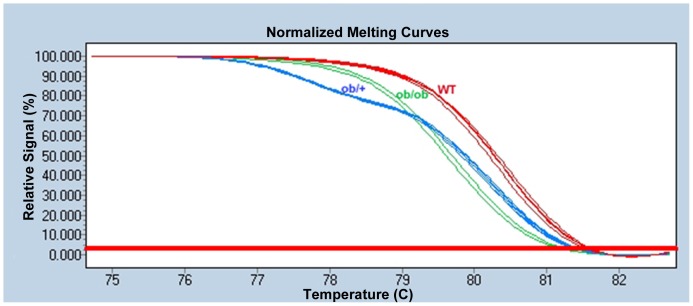
Fluorescence normalized high resolution melting curves of wild type (red), ob/ob (green), and ob/+ (blue) animals.

Fluorescence normalized high resolution melting curve plots clearly delineated the three genotypes (**Figure 2**). Homozygotes’ possessed sharply delineated Tm’s, with the homozygous ob/ob mutants 0.80±0.05 degrees lower than +/+ wild types due to the C-T transition which lowers their GC content (Cytosine forming 3 hydrogen bonds within the DNA duplex versus Thymine’s 2). The ob/+ heterozygote melting curve was much less steep, due to early melting of heteroduplex structures, thus producing a melting pattern distinct from both wild types and homozygotes. Furthermore, its Tm was 2.53±0.06 degrees lower than Wt and 1.73±0.08 below ob/ob homozygotes (all p values <0.0001).

### Phenotypic Confirmation of Genotypes

**Figure 3:**
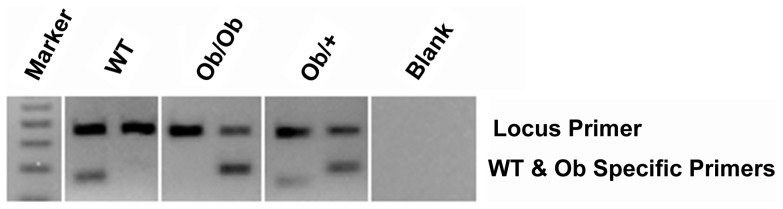
Confirmation of HRM results by alternate gel-based methods. Locus specific control band at 191^st^ lane and Lep^ob^ specifc band at 123bp in 2^nd^ lane of pairs.

**Figure 4:**
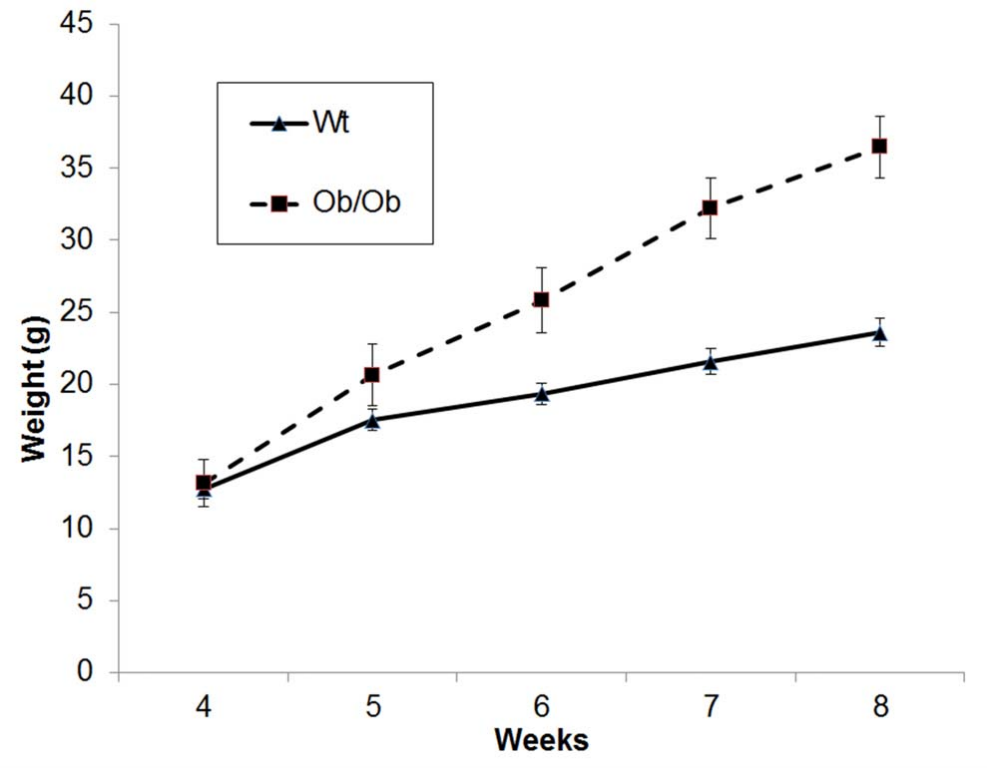
Weights of WT and Lep^ob^ animals from our breeding colonies genotyped by HRM method.

In ob/ob animals, weight gain can be used as a quantitative trait to confirm genotyping results. In addition to confirming the utility of our method in commercially procured ob/ob animals, we established ob/+ intercrosses and the resulting pups were genotyped using our previous gel-based method (**Figure 3**) and phenotyped to confirm HRM results [13]. As expected and previously described, homogygous ob/ob animals diverged in weight from their lean and heterozygous littermates at 5 weeks of age and continued to become significantly larger at 8 weeks (**Figure 4**).

## Discussion

**Figure 5:**
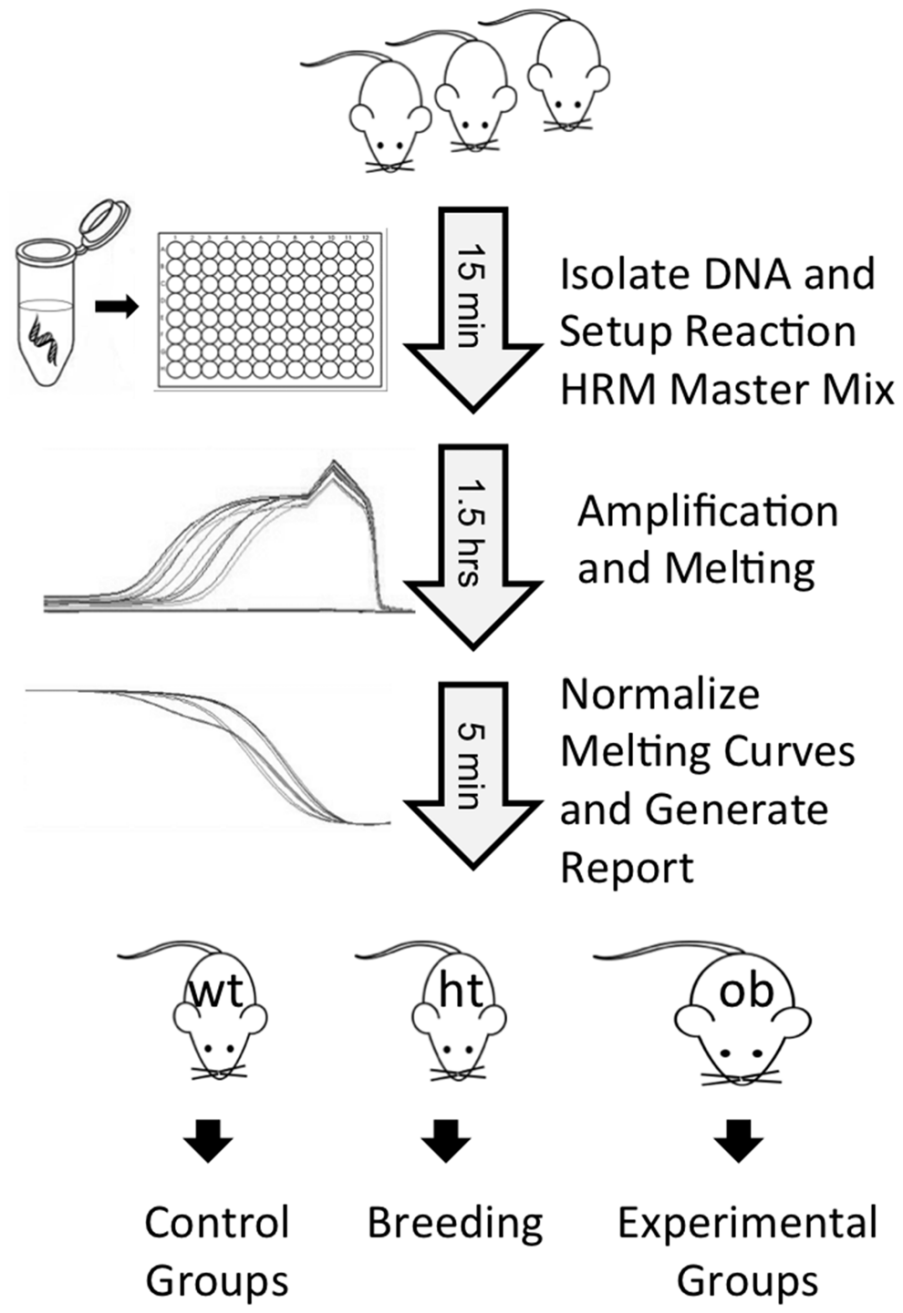
A simplified schematic of the HRM protocol.

Application of HRM to genotyping of the Lep^ob^ allele allows rapid genotyping of many animals in a 96 well format with the use of one PCR master mix. Thus, large colonies can be genotyped for this allele in little over 2 hours and with only 45 minutes of hands-on time (**Figure 5**). Interpretation and recording of results is simplified by the absence of gels, which must be imaged and archived separately from genotyping results. The assay is robust with respect to the quality and quantity of starting material. We have validated the method with DNA isolated using various kits and with unpurified lysate. Furthermore, the HRM method can distinguish between genotypes using a wide range of starting material concentrations. We have shown differentiation across a range from 0.30 to 30 ng of starting material, demonstrating the robustness of this assay with respect to template amount. However, one important feature of this system is that the product amounts be roughly equivalent between samples being compared within a run, as product concentrations can affect melting curves.

The primers and general method described here can be applied to HRM dyes and thermocyclers of different manufacture, such as Bio-Rad and ABI. Application of HRM genotyping to ob/ob colonies allows the rapid generation of multiple knockout models for the study of obesity and metabolic disease, whose global importance are of ever increasing urgency.
